# Network Analysis of Legg–Calve–Perthes Disease and Its Comorbidities

**DOI:** 10.3390/jcm14010259

**Published:** 2025-01-05

**Authors:** KyeongMi Kim, Kyung Rae Ko, Siyoung Yoon, Jaiwoo Chung, Soonchul Lee

**Affiliations:** 1 Department of Laboratory Medicine, CHA Ilsan Medical Center, CHA University School of Medicine, Goyang 10414, Republic of Korea; kmi0905@chamc.co.kr; 2 Department of Orthopaedic Surgery, Samsung Medical Center, Sungkyunkwan University School of Medicine, Seoul 06351, Republic of Korea; krmd.ko@gmail.com; 3 Department of Orthopaedic Surgery, CHA Bundang Medical Center, CHA University School of Medicine, Seongnam 13496, Republic of Korea; tldud1105@naver.com (S.Y.); jwc.os.cha@gmail.com (J.C.)

**Keywords:** Legg–Calvé–Perthes disease, comorbidity, big data, network analysis

## Abstract

**Background/Objectives**: Legg–Calvé–Perthes disease (LCPD) is characterized by idiopathic avascular necrosis of the femoral head in children. There are several hypotheses regarding the cause of LCPD; however, the exact cause remains unclear. Studies on comorbidities can provide better insight into the disease. We aimed to perform a network analysis to identify the associations between LCPD and comorbidities. **Methods**: We analyzed patients aged ≤ 12 years with LCPD as defined by ICD-10 codes M91.1, M91.8, and M91.9 registered in the National Health Insurance Service cohort database from 2002 to 2015. A control group was designed using propensity score matching. Comorbidities were identified and network analysis was performed. The identified comorbidities were reclassified into clinical disease groups considering their clinical relevance, and a network map was created using odds ratios. **Results**: In total, 23 significant disease clusters were identified. Injury-related disease clusters with ICD-10 codes starting with “S” were the most frequent. They were reclassified into 11 disease groups based on clinical relevance. Among these, congenital deformities of hip (Q65) had the highest odds ratio. Congenital deformities of feet (Q66) and other anemia (D64) had a single association with LCPD in the comorbidity network analysis. **Conclusions:** We confirmed the association between LCPD and comorbidities using a network analysis. The LCPD comorbidity network identified in this study is expected to serve as the basis for future research on LCPD.

## 1. Introduction

Many people suffer from diseases caused by a combination of individual susceptibility and repeated exposure to noxious environments or materials [1]. Furthermore, pre-existing diseases affect the occurrence of other diseases, and new diseases affect the course of pre-existing diseases. However, the degree to which these diseases interact is not well known. Hypothesis-driven epidemiological studies on disease interactions are conventionally used but are limited in that they are associated with a small population [2]. Data-driven research through network analysis using more integrated and larger datasets is being used to study multi-morbidity. In particular, network analysis is used to identify correlations with comorbidities in chronic diseases such as hypertension, diabetes, and chronic obstructive pulmonary disease [3,4]. Also, network analysis is mainly used in the field of psychiatric medicine, including pediatric autism [5], and has recently been used in the field of orthopedics to identity comorbidities related with osteoporosis [2]. Therefore, network analysis is widely applied extensively to describe, explore, and understand structural and relational aspects of health in medical field.

Legg–Calvé–Perthes disease (LCPD) is characterized by idiopathic avascular necrosis of the femoral head in children, with an incidence between 0.4 and 29.0 per 100,000 people [6,7]. LCPD was first described in 1910 [8]. Smoking habits and obesity are strongly associated with LCPD according to recent studies [9,10]. But its etiology is not fully known, although prenatal and environmental factors have been reported to increase its prevalence [6,11]. There is still controversy about the association of hypercoagulability, and potential genetic factors have also been studied [12,13,14]. LCPD in children younger than 6 years usually has a benign and self-limiting course, but older children may require surgical treatment and have increased risk of joint degenerative diseases, such as arthritis [8].

There are several studies on LCPD and comorbidities. Perry et al. reported in a case–control study that LCPD was significantly related to congenital genitourinary and inguinal anomalies [15]. A retrospective population-based cohort study by Hailer reported that obesity and hypothyroidism increased risk estimates in LCPD patients [16]. However, most studies are on the association between a single or a small number of diseases and LCPD [15,16]. Further studies on the link between LCPD and other diseases using big data and network analysis can provide valuable insights into their interactions and pathogenesis. Therefore, we aimed to perform a network analysis, a relatively novel research method, to identify novel or poorly known associations between LCPD and other comorbidities using a nationwide Korean database.

## 2. Materials and Methods

### 2.1. Data Source

Our analysis data were obtained from health insurance claims data in South Korea. Health care in South Korea is supplied by National Health Insurance (NHI), which is mandatory and required by Korean law. The National Health Insurance Service (NHIS) National Sample Cohort (NSC) database contains the demographic information of each patient and history of medical treatment, including the diagnosis code. We analyzed the records of about 1 million out of 50 million Korean individuals (approximately 2%).

The database used in this study contained de-identified personal information about patients from 2002 to 2015. Diseases were coded according to the 10th revision of the International Statistical Classification of Diseases and Related Health Problems (ICD). This study has been approved by the Institutional Review Board (IRB) of CHA University Bundang Hospital, Korea (IRB No. CHAMC 2020-08-004).

### 2.2. Incidence

An incident case was defined as a person newly diagnosed and registered as an LCPD patient in the corresponding year. We selected 1 year (2002) as the washout period. The incidence rate of LCPD was expressed as the number of new cases per 100,000 people. We confirmed the sex-related differences in the incidence of LCPD.

### 2.3. LCPD Group

**Figure 1:**
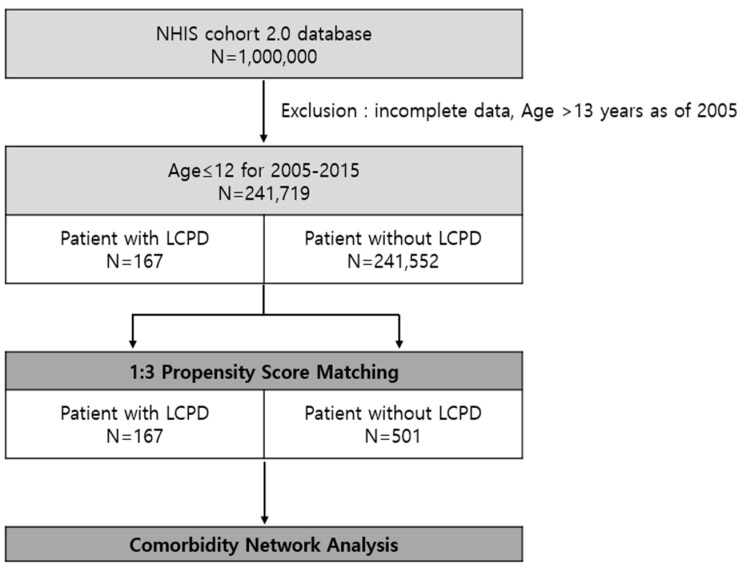
Study population. We found 167 patients diagnosed with Legg–Calvé–Perthes disease (LCPD) and 241,552 patients that have never been diagnosed with LCPD in the Korea NHIS cohort database.

We followed a systematic process of cleaning, filtering, and creating a suitable cohort to distinguish the research dataset. Initially, we excluded patients with incomplete data. For example, we excluded patients without basic demographic data and who did not visit the hospital during the observational period. We included patients aged ≤ 12 years at the initial visit with ICD-10 of M91.1 (juvenile osteochondrosis of head of femur), M91.8 (other juvenile osteochondrosis of hip and pelvis), and M91.9 (juvenile osteochondrosis of hip and pelvis, unspecified). Next, a 3-year washout period (2002–2004) was used to limit the group to patients who were first diagnosed with LCPD, thus limiting the possibility that the first event was a readmission or follow-up treatment. Finally, 167 patients were selected from the LCPD group (Figure 1).

### 2.4. Control Group

We used propensity score matching (PSM) to compare the case and control groups, which reduces possible bias originating from the difference in patient demographic characteristics to adjust for potential confounding variables [17]. LCPD was considered as the dependent variable, while baseline characteristics such as age, sex, and period before diagnosis were considered as pre-defined covariates. The period before the diagnosis is a new variable that minimizes the impact of missing information. Using this variable as a covariate for PSM, we matched the period of medical records prior to the diagnosis of case patients to that of control patients. For example, if a patient had 3 years of medical records prior to diagnosis, a matched control patient had the same for the period. We extracted 241,552 patients without LCPD for the candidates of the control group. The patients in the case group (with LCPD) were based on a 1:3 fixed ratio using a greedy algorithm of the nearest-neighbor matching method, which is commonly used in epidemiology, rather than an artificial intelligence algorithm.

### 2.5. Disease Classification

We utilized ICD-10 codes to identify the comorbidities in the study and control groups. An ICD-10 code consists of three characters, the first of which is a letter identifying the “chapters”. The ICD-10 is organized into 22 chapters that are subdivided into homogeneous blocks of three-character categories. Each of these three-character categories represent a single condition. The ICD-10 code can be made more specific by adding the fourth and fifth numeric characters. The fourth and fifth characters serve to further subdivide the etiology, anatomic site, or severity of each category according to each situation. We found 1515 unique ICD-10 codes in our cohort. However, would have been impractical to run analyses using these diagnostic codes individually. To simplify the study, we truncated the ICD-10 codes beyond their third digit to reduce the number of diseases coded to 474. Furthermore, we extracted patient medical records prior to the initial visit for the diagnosis of LCPD. The control group was extracted based on the data from the case group. After this process, we recorded 345 unique ICD-10 codes.

### 2.6. Analysis of Association

From the 345 distinct comorbidities in the case group, we determined the association of every possible link among all comorbidities by calculating the odds ratio using the Haldane–Anscombe correction to add 1/2 to all cells of the contingency (2 × 2) table to circumvent the zero-cell problem [18,19]. For each pair of ICD-10 codes, we performed Fisher’s exact tests to determine whether the two codes were significantly associated, which were considered significant if the *p*-value was <0.05. We found 23 comorbidities, which passed Fisher’s exact test for *p*-value <0.05. We reclassified into 11 disease groups based on their clinical relevance from the 23 disease groups and further analyzed them using grouped data.

### 2.7. Network Map

The network was constructed using LCPD and its 11 disease groups. Each comorbidity was represented in a graph by a specific node with two attributes. The diameter of the nodes was measured by their connectivity. The degree referred to the number of connections among disease clusters (the number of links connected to a node). For example, if LCPD had 11 degrees, it meant that LCPD had 11 linkages with other disease clusters. The color of the nodes represents the chapter of the ICD-10 code [20]. That is, the color was assigned according to the first letter of the ICD-10 code. Edges connecting nodes represent statistically significant associations. Edge thickness represents the strengths of association (odds ratio).

### 2.8. Statistical Analysis

All analyses were performed using SAS EG software (version 7.1; SAS Institute Inc., Cary, NC, USA) and free R software (version 3.6.2; R Development Core Team, Auckland, New Zealand). We used Gephi Graph visualization and manipulation software (version 0.9.2, WebAtlas, Paris, France) to create network graphics [21].

## 3. Results

### 3.1. Incidence in Korean Population

**Figure 2:**
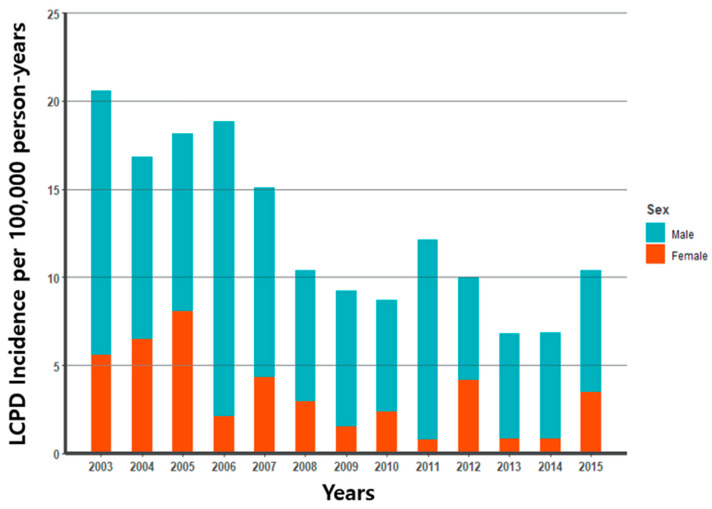
Legg–Calvé–Perthes incidence (per 100,000) for 2003–2015.

Analysis using the NHIS-NSC database of South Korea showed a decreasing trend in the incidence rate of LCPD in children aged ≤ 12 years from 2003 to 2015. The mean annual incidence for the entire cohort is 9.3/100,000 in boys and 3.4/100,000 in girls (*p*-value < 0.001). We observed a higher incidence of LCPD in boys than in girls during all periods. In boys, the incidence rate of LCPD was highest (16.7/100,000) in 2006 and lowest (5.8/100,000) in 2012 compared to our other analysis years. Incidence among girls was highest with 8.0 in 2005 and lowest with 0.8 in 2011 (Figure 2).

### 3.2. Study Population

In total, 241,719 patients ≤12 years of age were registered in the database between 2005 and 2015. Of these, 167 were diagnosed with LCPD. A control group of 501 patients was selected by PSM with the case group based on their characteristics. After matching the covariates, there were no statistically significant differences between the case and matched groups for any of the matching covariates (Table 1).

### 3.3. Comorbidity Network

Among the 345 diseases identified through the NHIS-NSC records before LCPD diagnosis, 23 comorbidities showed statistically significant differences between those diagnosed with LCPD and those without LCPD (Table 2). Injury-related disease clusters with ICD-10 codes starting with “S” were the most frequent (7/23). In addition, there were six disease clusters starting with “M” related to musculoskeletal system and connective tissue disease. Based on the odds ratio, the top diseases associated with LCPD were congenital deformities of hip (Q65), congenital deformities of feet (Q66), pyogenic arthritis (M00), other disorders of synovium and tendon (M67), and scoliosis (M41).

**Figure 3:**
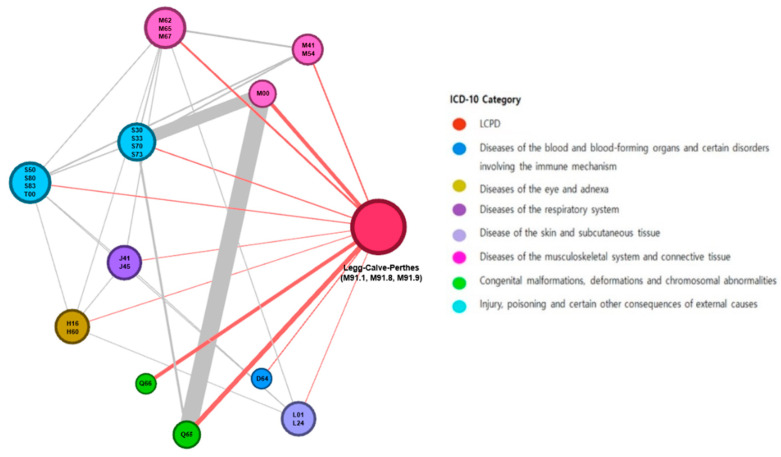
Legg–Calvé–Perthes disease and the comorbidity network. The sizes of the nodes are proportional to the degree (number of links to other disease clusters). The colors of the nodes are the ICD-10 chapters. The edge thickness is related to the odds ratio value between diseases.

We reorganized the 23 disease clusters into 11 disease groups considering their clinical relevance (Table 3). The odds ratio for congenital deformities of hip (Q65) was the highest at 27.61. Congenital deformities of feet (Q66) and pyogenic arthritis (M00) had odds ratios of 21.34 and 21.34, respectively. In addition, we generated a network map of comorbidities (Figure 3), and found that congenital deformities of feet (Q66) and other anemia (D64) had a single association with LCPD, but other disease clusters were abundantly linked between LCPD and other disease clusters, as well as between the other disease clusters themselves.

## 4. Discussion

We assessed the comorbidities related to LCPD in children aged ≤ 12 years and visualized them as a network. Through a data-driven investigation, we obtained a comprehensive understanding of comorbidities, which allowed us to analyze the relative importance of each disease association. Additionally, we identified illnesses that are already known to be associated with LCPD, as well as some lesser-known associations.

In the pediatric population, LCPD was formerly considered to be osteochondrosis, but it is now known as idiopathic avascular necrosis of the femoral head. There are also significant differences in incidence rates by country, city, and racial groups [6]. The incidence in Caucasians has been reported to range from 4.4 to 16.9 per 100,000 in England and from 5.7 to 10 in the United States; in Japanese children, 0.9/100,000; and in Hong Kong, 0.2/100,000 [7]. In a study of Korean children from 1999 to 2001, the mean annual incidence was 3.8/100,000 [22]. In our study using big data from the NHIS-NSC database, the average incidence of Korean children from 2003 to 2015 was 9.3 in boys and 3.4 in girls, showing a nearly 3:1 ratio with a greater incidence in boys. The incidence in our study was lower than that in England but higher in the same Asian population than in Japan or Hong Kong.

The explanation for the differences in LCPD incidence by country, region, and race remains unclear. Causes have been hypothesized as anatomical differences in arterial supply to the femoral head, socio-economic variables, differences in skeletal maturation, and genetic factors [23,24,25,26]. We aimed to gain a better understanding of the etiology of LCPD by examining the association between LCPD and diseases that occurred prior to being diagnosed with LCPD.

We investigated the records of children ≤ 12 years of age who visited hospital prior to being diagnosed with LCPD using the NHIS-NSC database. A total of 345 ICD-10 codes were found in patients diagnosed with LCPD; among them, 23 statistically significant disease clusters were identified. In particular, the odds ratio was the highest at 27.61 in congenital anomaly of hip (Q65) and 21.34 in congenital anomaly of feet (Q66). Another study has also reported that the incidence of LCPD in patients with developmental dysplasia of the hip (DDH) is higher than in the normal population [24]. The incidence of LCPD increases in abnormal birth presentation, and the same risk factor exists in DDH [24,27]. In addition, a case of Bardet–Biedl syndrome (BBS) harboring an MKKS/BBS6 mutation with polydactyly of the toes and LCPD has also been reported. It has also been reported that children with LCPD have a higher incidence of congenital anomalies than controls, supporting the results of our analysis [28].

LCPD progresses through the course of hip synovitis, femoral epiphysis condensation and fragmentation, re-ossification, and remodeling. Inflammation plays a fundamental role in the disease as significant and persistent synovitis is present throughout the course of the disease [29]. In many studies, increased levels of the pro-inflammatory cytokine interleukin-6 (IL-6) have been observed in the synovial fluid of patients with LCPD [30]. The odds ratio for pyogenic arthritis (M00) was also observed to be as high as 21.34 in our research. This result is thought to be due to secondary LCPD caused by septic arthritis, corticosteroids, and so on.

The group with other disorders of muscle (M62), synovitis and tenosynovitis (M65), and other disorders of synovium and tendon (M67) had an odds ratio of 10.23, and scoliosis (M41) and dorsalgia (M54) showed an odds ratio of 6.63. These diseases showed high odds ratios, and it is possible to consider the possibility that pain, limitation of motion, and limp, which are characteristics of LCPD, were also observed in these diseases or occur secondary to them. We also confirmed that other anemias (D64) had an odds ratio of 3.51. Among the pathophysiologies of LCPD, it is hypothesized that the blood supply to the femoral head is insufficient due to vascular pathology. According to a study by Hailer et al., the risk of anemia is higher in patients with LCPD [31], which was consistent with our analysis. However, further research, including animal experiments, is needed to determine the clear pathophysiology of the hematological involvement.

## 5. Study Limitations

Our study may provide new insights into the etiology or pathogenesis of LCPD. However, this study has several limitations. First, the registered ICD-10 codes may be either inaccurate or subjective. The code is typically used for administrative and billing purposes. The probability that the practitioner used an inaccurate ICD-10 code cannot be ruled out. Second, we used only three characters of the ICD-10 codes. The fourth and fifth characters in the ICD-10 codes were used to further refine the etiology, anatomical location, or severity of each category. In future, this information will be included in the analysis, and it is expected that more accurate and significant disease association results will be obtained. Third, the dataset used in this study does not distinguish between primary and secondary LCPD. Therefore, there are limitations in confirming the cause and effect between primary LCPD and comorbidities. Forth, some cases might have had preliminary or temporary diagnoses before they were finally diagnosed with LCPD; therefore, the possibility that it may have been misunderstood as comorbidities cannot be ruled out. Nevertheless, our analysis is meaningful because it provides a global overview using much real-world data based on the NHIS-NSC database. Moreover, the advancement of artificial intelligence and machine learning methods, as well as network analysis, are expected to allow future studies to better explore these diseases.

## 6. Conclusions

In conclusion, we confirmed the association between LCPD and comorbidities using network analysis. Using this approach, we identified an association that could help provide new strategies to approach the disease, although we still do not know the clinical implications of these associations. The global view of the LCPD comorbidity network presented in this study is expected serve as a basis for future studies on LCPD.

## Figures and Tables

**Table 1:** Covariates of patients for propensity score matching.

Variables	Propensity Score Matched (1:3)		*p*-Value
	LCPD (N = 167)	Non-LCPD (N = 501)	
Sex			1 *
Male	125 (74.9%)	375 (74.9%)	
Female	42 (25.1%)	126 (25.1%)	
Age (years)	7.8 ± 2.7	7.8 ± 2.7	1 †
Period before diagnosis (years)	5.1 ± 2.3	5.1 ± 2.3	1 †

LCPD, Legg–Calvé–Perthes disease. * Pearson’s chi-square test, † *T*-test.

**Table 2:** Odds ratio (OR) and degree in LCPD comorbidity network.

ICD-10 Code	English Name	LCPD N (%)	Non-LCPD N (%)	*p*-Value	OR	Degree
D64	Other anaemias	8 (4.8)	7 (1.4)	0.016	3.51	3
H16	Keratitis	30 (18)	59 (11.8)	0.048	1.65	4
H60	Otitis externa	43 (25.7)	85 (17)	0.017	1.7	4
J41	Simple and mucopurulent chronic bronchitis	17 (10.2)	22 (4.4)	0.012	2.48	4
J45	Asthma	124 (74.3)	330 (65.9)	0.045	1.49	4
L01	Impetigo	62 (37.1)	139 (27.7)	0.025	1.54	8
L24	Irritant contact dermatitis	40 (24)	76 (15.2)	0.013	1.77	5
M00	Pyogenic arthritis	3 (1.8)	0 (0)	0.015	21.34	4
M41	Scoliosis	4 (2.4)	1 (0.2)	0.015	9.18	5
M54	Dorsalgia	7 (4.2)	4 (0.8)	0.007	5.17	7
M62	Other disorders of muscle	7 (4.2)	4 (0.8)	0.007	5.17	6
M65	Synovitis and tenosynovitis	15 (9)	8 (1.6)	<0.001	5.9	5
M67	Other disorders of synovium and tendon	40 (24)	12 (2.4)	<0.001	12.44	9
Q65	Congenital deformities of hip	4 (2.4)	0 (0)	0.004	27.61	2
Q66	Congenital deformities of feet	3 (1.8)	0 (0)	0.015	21.34	1
S30	Superficial injury of abdomen, lower back and pelvis	5 (3)	4 (0.8)	0.048	3.74	6
S33	Dislocation, sprain and strain of joints and ligaments of lumbar spine and pelvis	15 (9)	11 (2.2)	<0.001	4.34	11
S50	Superficial injury of forearm	10 (6)	12 (2.4)	0.041	2.61	2
S70	Superficial injury of hip and thigh	10 (6)	7 (1.4)	0.003	4.4	8
S73	Dislocation, sprain and strain of joint and ligaments of hip	16 (9.6)	10 (2)	<0.001	5.1	6
S80	Superficial injury of lower leg	16 (9.6)	19 (3.8)	0.008	2.7	10
S83	Dislocation, sprain and strain of joints and ligaments of knee	16 (9.6)	15 (3)	0.001	3.42	10
T00	Superficial injuries involving multiple body regions	8 (4.8)	7 (1.4)	0.016	3.51	1

**Table 3:** Odds ratio (OR) and degree in LCPD comorbidity network after grouping.

ICD-10 Code	English Name	LCPD N (%)	Non-LCPD N (%)	*p*-Value	OR	Degree
D64	Other anaemias	8 (4.8)	7 (1.4)	0.016	3.51	1
H16	Keratitis	60 (35.9%)	130 (25.9%)	0.017	1.6	5
H60	Otitis externa					
J41	Simple and mucopurulent chronic bronchitis	129 (77.2%)	336(67.1%)	0.017	1.65	5
J45	Asthma					
L01	Impetigo	79 (47.3%)	185 (36.9%)	0.022	1.53	5
L24	Irritant contact dermatitis					
M00	Pyogenic arthritis	3 (1.8)	0 (0)	0.015	21.34	3
M41	Scoliosis	11 (6.6%)	5 (1.0%)	<0.001	6.63	4
M54	Dorsalgia					
M62	Other disorders of muscle	54 (32.3%)	22 (4.4%)	<0.001	10.23	7
M65	Synovitis and tenosynovitis					
M67	Other disorders of synovium and tendon					
Q65	Congenital deformities of hip	4 (2.4)	0 (0)	0.004	27.61	3
Q66	Congenital deformities of feet	3 (1.8)	0 (0)	0.015	21.34	1
S30	Superficial injury of abdomen, lower back and pelvis	32 (19.2%)	25 (5.0%)	<0.001	4.48	6
S33	Dislocation, sprain and strain of joints and ligaments of lumbar spine and pelvis					
S70	Superficial injury of hip and thigh					
S73	Dislocation, sprain and strain of joint and ligaments of hip					
S50	Superficial injury of forearm	42 (25.1%)	48 (9.6%)	<0.001	3.17	7
S80	Superficial injury of lower leg					
S83	Dislocation, sprain and strain of joints and ligaments of knee					
T00	Superficial injuries involving multiple body regions					

## Data Availability

Data are contained within the article.
